# Shoulder Stretching Intervention Reduces the Incidence of Shoulder and Elbow Injuries in High School Baseball Players: a Time-to-Event Analysis

**DOI:** 10.1038/srep45304

**Published:** 2017-03-27

**Authors:** Hitoshi Shitara, Atsushi Yamamoto, Daisuke Shimoyama, Tsuyoshi Ichinose, Tsuyoshi Sasaki, Noritaka Hamano, Akira Ueno, Fumitaka Endo, Atsufumi Oshima, Hideo Sakane, Masahiro Tachibana, Yusuke Tomomatsu, Tsuyoshi Tajika, Tsutomu Kobayashi, Toshihisa Osawa, Haku Iizuka, Kenji Takagishi

**Affiliations:** 1 Department of Orthopedic Surgery, Gunma University Graduate School of Medicine, 3-39-22 Showa, Maebashi, Gunma 371-8511, Japan; 2 Department of Physical Therapy, Takasaki University of Health and Welfare, 37-1 Nakaorui, Takasaki, Gunma 370-0033, Japan; 3 Department of Orthopedic Surgery, National Hospital Organization Takasaki General Medical Center, 36 Takamatsu, Takasaki, Gunma 370-0829, Japan; 4 Department of Orthopedic Surgery, Saint Pierre Hospital, 786-7 Kamisano, Takasaki, Gunma 370-0857, Japan

## Abstract

We prospectively evaluated the effects of a prevention program on the incidence of shoulder and elbow injuries in high school baseball pitchers. Ninety-two pitchers participated in this study and were taught to perform stretching and strength exercises aimed at improving shoulder external rotation strength in the preseason. The pitchers freely chose to participate in one of four groups [SM-group: performed both exercises, S-group: performed stretching exercise only, M-group: performed strength training only, and N-group: performed neither intervention]. Injury was defined as inability to play for ≥8 days because of shoulder/elbow symptoms. Kaplan-Meier survival curves were generated and hazard ratios (HRs) for injury occurrence were calculated using multivariate Cox regression. Log-rank test was used for between-group comparisons of survival distributions. The injuries occurred in 25, 35, and 57% of participants and median times to injury were 89, 92, and 29.5 days in the S- (n = 32), SM- (n = 46), and N- (n = 14) group, respectively. Nobody chose M-group. HRs were 0.36 and 0.47 for the S- and SM-group, respectively, based on the N-group. The incidence of injury was significantly lower in the S-group than in the N-group (*p* = 0.04). Daily posterior shoulder stretching may reduce the incidence of the injuries in high school baseball pitchers.

Shoulder and elbow injuries are common in baseball pitchers. Few prospective studies have investigated the risk factors for throwing-related shoulder and elbow injuries in the preseason. Among high school pitchers, Shitara *et al*. have shown that risk factors for preseason injury include a low dominant to nondominant side prone external rotation ratio and decreased dominant side passive 90° abducted internal rotation range of motion (ROM)^1^. Among high school baseball and softball players, risk factors have been shown to include shoulder internal rotation and horizontal adduction ROM deficits^2^. For comparison, risk factors for shoulder injury in professional baseball pitchers include weak external rotation and poor supraspinatus strength^3^.

Although risk factors for injury to baseball pitchers should be targeted for preventative interventions, no studies have investigated the outcomes of upper body injury prevention programs^4^. There is a clear need to investigate if interventions targeted to specific injury risk factors can reduce the incidence of shoulder and elbow injuries in baseball players. Furthermore, to prevent injuries it is also important to determine when injuries occur during the season. Thus, we prospectively evaluated the effects of an injury prevention program consisting of a shoulder stretching exercise and strength training on the incidence of shoulder and elbow injuries in high school baseball pitchers. A prior prospective study of a homogenous group of high school pitchers showed that deficits in internal rotation on the dominant side and weakness of prone external rotation strength were risk factors for injury^1^; therefore, we designed our prevention program to improve both internal rotation ROM and prone external rotation strength.

## Results

### Baseline characteristics

All 92 pitchers were followed without drop-out. There were 14, 32, and 46 pitchers in the N-, S-, and SM-group, respectively. No pitcher selected only strength training. The mean adherence to the prevention program in the S- and SM-group was 77 and 65%, respectively. In the preseason baseline assessment, there were no significant between-group differences in baseball experience, height, weight, 90° abducted shoulder internal rotation ROM, horizontal adduction in the dominant shoulder, prone external and internal rotation strength in the dominant shoulder, prone external and internal rotation strength ratios, and elbow flexion and extension on the dominant side (Table 1). Thus, during the preseason, the participants in each group had the same risk of experiencing shoulder and elbow injuries during the season.

### Time-to-event-analysis

**Figure 1:**
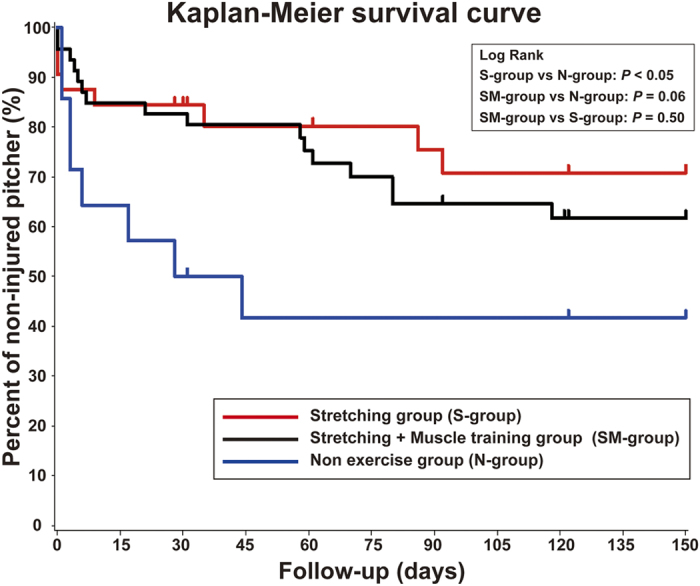
Kaplan-Meier survival curves. The median survival times were 89, 92, and 29.5 days in the S-, SM-, and N-group, respectively. A log-rank test showed that injury incidence was significantly lower in the S-group than in the N-group (*p* = 0.04). There were no significant differences between the S- and SM-group (*p* = 0.50), or between the SM- and N-group (*p* = 0.06).

The incidences of shoulder and/or elbow injuries in the S-, SM-, and N-group were 8 (25%), 16 (35%), and 8 (57%), respectively. The median survival times were 89, 92, and 29.5 days in the S-, SM-, and N-group, respectively (Fig. 1). Kaplan-Meier analysis yielded HRs of 0.36 and 0.47 in the S- and SM- group, respectively, based on the N-group (Table 2). A log-rank test showed that injury incidence was significantly lower in the S-group than in the N-group (*p* = 0.04). There were no significant differences between the S- and SM-group (*p* = 0.50), or between the SM- and N-group (*p* = 0.06; Fig. 1).

### *Post-hoc* power analysis

*Post-hoc* power analysis showed that the statistical powers were 0.85, 0.68, and 0.19 between the N- and S-group, between the N- and SM-group, and between the S- and SM-group, respectively.

## Discussion

The most important finding of the present study was that the self-stretching exercise significantly reduced the incidence of shoulder and elbow injuries in high school baseball pitchers. Furthermore, our results demonstrated that pitchers who performed daily sleeper stretching were able to continue pitching during the season approximately three times longer than pitchers who did not perform daily sleeper stretching. This is the first prospective study to provide evidence that a prevention program designed to address known risk factors for shoulder and elbow injuries can effectively prevent those injuries.

### Self-stretching exercise for posterior shoulder tightness

Decreased passive internal rotation of the dominant shoulder at 90° abduction during the preseason has been previously shown to be a risk factor for injury in high school baseball players^1^,^2^. Thus, in this study, we used sleeper stretching as an intervention aimed at correcting internal rotation deficits due to posterior shoulder tightness. The reduced number and later onset of injuries reported by pitchers who participated in the stretching program might be because of improved internal rotation ROM related to internal and subacromial impingement^5^,^6^, increased posterior shoulder flexibility^7^, and/or increased acromiohumeral distance, which is related to glenohumeral internal rotation deficit (GIRD)^8^. Although we did not measure shoulder ROM and acromiohumeral distance during the season in this study, we have previously shown an association between dominant shoulder internal rotation and the incidence of shoulder injury with an adjusted odds ratio, calculated by logistic regression analysis, of 0.95^1^. In other words, a 9° increase in dominant shoulder internal rotation resulted in a 36% reduction of the risk of injury (calculated odds ratio = 0.64). This estimated 36% risk reduction corresponds with the magnitude of the prevention effect of sleeper stretching seen in this study; it differs slightly from the adjusted odds ratio calculated by logistic regression analysis and HR calculated by multivariate Cox regression because these analyses do not consider time factors.

In this study, more stretching (five repetitions per day of 60 seconds of stretching) was performed than in previous studies (three repetitions per day of 30 seconds of stretching)^7^,^8^. Moreover, we instructed participants to perform the preventative exercises after daily baseball practice and not as part of their warm-up routine to reduce the tightness produced by pitching and to reduce variability in stretching performance. The timing and amount of stretching might affect shoulder and elbow injury incidence during the season. Future studies should assess what amount and timing of stretching are needed to most effectively prevent shoulder and elbow injuries in high school pitchers.

### Strength training

Muscle strength training to improve shoulder external rotation was included in our prevention program because previous studies have shown that a preseason decrease in the ratio of dominant to nondominant side prone external rotation is a risk factor for injury in high school and professional baseball pitchers^1^,^3^. In this study, no participants chose to only perform strength training. Thus, future studies should assess the effectiveness of strength training for improving shoulder external rotation and preventing shoulder and elbow injuries in high school pitchers.

In this study, the combination of sleeper stretching and strength training tended to reduce the incidence of injuries (*p* = 0.06), and there was no significant difference in injury incidence between sleeper stretching and the combination of sleeper stretching and strength training (*p* = 0.50). Although this incidence reduction in the combination of sleeper stretching and strength training group compared with non-prevention group was not significant, we believe that stretching combined with strength training may prevent throwing-related injuries because post hoc power analysis demonstrated that our study had insufficient power to detect differences between the N- and SM-group.

Although we could have increased the number of participants by pooling data from several years or collecting data from several prefectures, we intentionally chose not to in the current study. We collected data from a single prefecture to reduce variety in time-related factors (e.g., the beginning the season, timing of major games, long practice interruptions due to weather conditions) that might have affected our time-to-event analysis. In addition, we chose to collect data from a single prefecture in which we have an excellent relationship with the high school baseball federation and have been performing annual medical check-ups for baseball players during prefectural tournaments for the past 10 years. Selection of this prefecture insured that our data would be high quality and reliable, and participants were able to communicate with study coordinators in a timely and consistent manner (every 2–4 weeks).

The use of a standardized muscle strength training protocol (load approximately 500 g, 20 repetitions/set, 3 sets/day) may have affected our results, that the incidence rate in in the combination of sleeper stretching and strength training group seems to be higher than that in the only sleeper stretching group and the incidence reduction in the combination of sleeper stretching and strength training group compared with non-prevention group was not significant, because each participant had different baseline shoulder external rotation strength. Adjusting the load and frequency based on preseason strength might have led to different results. Future studies should assess the appropriate load and frequency of strength training exercises to reduce or prevent shoulder and elbow injuries in high school pitchers.

Although no previous studies have attempted to prevent pitching-related injuries through strength training, Edwards *et al*. reported that a combination of nonsteroidal anti-inflammatory drugs, posterior capsular stretching, and a strengthening program significantly improved pain, function, and quality of life in patients with anterior posterior tears of the superior labrum^6^. Even though the combination of sleeper stretching and strength training did not achieve significant reduction of the injury incidence in this study, the combination of sleeper stretching and strength training used in this study might prevent pain and maintain shoulder function for pitching if this study had sufficient power to detect differences compared with control.

### Limitations

This study had several limitations. First, during group selection we allowed the participants to decide if they would perform stretching and/or strength training, and asked them to continue performing their selected program every day without any information regarding the program objectives. Thus, it is possible that this study includes some self-selection bias that may have affected the results. However, we did not find it ethically appropriate to randomly assign the participants to the prevention or non-prevention groups because of the potential for increased risk of injury to the non-prevention group. Even though there might have been some self-selection bias, there were no significant baseline differences among the groups. Also, we hypothesize that baseball pitchers require greater awareness of their physical condition than position players because the load on their shoulders and elbows is greater. Second, sample selection bias might exist because we collected data from participants in a single prefecture. However, this intervention had not been previously performed, so the participants were naïve to the prevention program; thus, we believe that limiting our data collection to one prefecture would not have affected the results. Third, we do not know the relationship between the time course of changes in ROM and shoulder strength and the time-to-event analysis in each group because we did not measure the time course of these changes during the season. This relationship should be investigated in future studies.

## Conclusion

Sleeper stretching significantly prevented baseball-related shoulder and elbow injures and significantly prolonged pitching availability during the season. The results of this study provide the first evidence of the efficacy of an injury prevention program aimed at reducing baseball-related shoulder and elbow injures in high school pitchers.

## Methods

### Participants

One hundred and thirty-two male high school baseball pitchers who participated in an annual preseason medical check-up during January and February 2013 were asked to enroll in this study. Ninety-two pitchers ranging in age from 15–17 years old agreed to participate. Forty pitchers did not enroll in this study because we did not complete the process of the taking informed consent or they refused participation. Inclusion criteria were that each pitcher: (1) participated in preseason workouts as an active pitcher, and (2) had no restrictions on their pitching activity. We excluded pitchers from this study if they: (1) had a prior injury (e.g., fracture) to the throwing arm, (2) were unable to throw or had restricted pitching activity because of a shoulder or elbow problem, or (3) performed daily muscle-specific rotator cuff training exercises or posterior capsule/sleeper stretches except for team practice.

This study was approved by the Institutional Review Board of Gunma University Hospital (Identification number 1003). All methods were carried out in accordance with relevant guidelines and regulations. Informed consent was obtained from the participants and their parents.

### Medical check-ups

Each participant underwent a baseline medical examination that evaluated the preseason condition of their shoulders and elbows. Examiners were blinded to the participants’ hand dominance. The following parameters were evaluated: (1) baseball experience, (2) height and weight, (3) shoulder and elbow ROM, and (4) shoulder muscle strength.

#### Shoulder and elbow ROM measurements

As previously reported^1^, all shoulder ROM data were measured by a single certified orthopedic surgeon using a digital protractor (iGaging, CA, USA). We have previously established the intrarater validity and reliability of the goniometer and hand-held dynamometers^1^,^9^. The passive ROM of shoulder internal rotation at 90° of abduction and horizontal adduction were determined for the dominant and nondominant shoulders using an examination table, and a digital goniometer with a bubble level was used to measure shoulder ROM^1^,^2^,^10^,^11^. For the measurements, the pitchers were placed in a supine position with their humerus abducted to 90°. To measure 90° abducted shoulder internal rotation, the humerus was kept parallel to the floor using a small towel roll under the elbow. The examiner used his thenar eminence and thumb to apply a posterior force through the coracoid process to stabilize the scapula before the arm was rotated^1^,^2^,^10^,^12^, and the humerus was then passively rotated at the end of 90° abducted internal rotation with the force of gravity acting on the arm. To measure horizontal adduction, the pitcher was placed with their elbow flexed to 90° and the scapula was stabilized behind the chest wall. The humerus was then moved passively into horizontal adduction. Shoulder ROM measurements were obtained by the examiner while an assistant provided a stabilizing force to maintain the shoulder position^13^. Elbow flexion and extension ROM were also passively measured while the participants lay in a supine position. ROM measurements were performed before muscle strength measurements because muscle tonus can vary with the effects of reciprocal inhibition due to muscle contraction.

#### Shoulder muscle strength measurements

A single certified orthopedic surgeon performed isometric strength measurements according to a standardized protocol similar to that described in previous studies^1^,^3^. Quantitative muscle strength during prone internal and prone external rotation in both the dominant and nondominant shoulder was assessed using a PowerTrack II Commander hand-held dynamometer (J-Tech Medical, Salt Lake City, UT, USA). Three trials were performed for each muscle strength measurement, and the median value from the three trials was recorded and used in subsequent analyses. Prone external rotation strength was measured with each participant lying prone on an examination table with their humerus abducted to 90° in the coronal plane and their elbow flexed to 90°. With the humerus manually stabilized by the examiner and the arm at 0° rotation, the dynamometer was placed on the dorsal aspect of the forearm 5 cm proximal to the proximal wrist extension crease. The participant was then instructed to rotate their arm externally with maximum power. Prone internal rotation strength was measured in a similar manner; however, the dynamometer was placed on the volar aspect of the distal radius 5 cm proximal to the proximal wrist flexion crease as the participant internally rotated their arm with maximum power. The dominant to nondominant ratios of prone external and internal rotation strength (prone external and internal rotation strength ratios) were calculated for each pitcher and used in the subsequent statistical analyses.

### Prevention program

**Figure 2:**
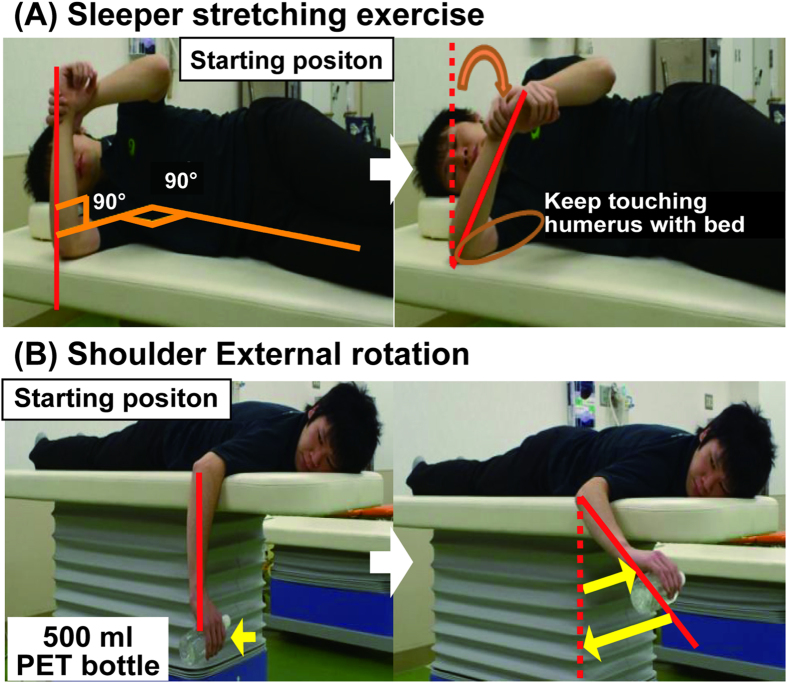
Injury prevention program. (**A**) Sleeper stretching exercise. (1) The subject lies in a right lateral position with their right scapula in contact with the bed, (2) the dominant shoulder is flexed to 90°, the dominant elbow is flexed to 90°, and the dominant forearm is in a neutral starting position, (3) the right forearm is grasped proximal to the dominant wrist with the non-dominant hand, (4) the dominant shoulder is passively internally rotated to the end of its maximum range of motion and that position is held for 1 minute (repeat 5 times). (**B**) Shoulder external rotation strength (1) The subject lies prone on the bed with their dominant shoulder 90° abducted, dominant elbow flexed at 90°, and dominant forearm in a neutral starting position, while grasping a 500-ml plastic bottle filled with water (weight: approximately 500 g), (2) the dominant shoulder is rotated externally for 1 second (concentric exercise) and then returned to the starting position over 1 second (eccentric exercise). This is repeated 20 times per set. (3) After a short break the set is repeated to a total of three sets.

During the preseason, each participant received 30 min of one-on-one instruction in self-stretching exercises and strength training from an experienced physical therapist. Participants also received a brochure with illustrations of the exercises, a written summary of the instructions, and an overview of compensatory motions to avoid (Fig. 2). During the one-on-one instruction, the focus was on correct performance of the exercises and avoiding compensatory movements. To avoid selection bias, we did not give the participants any information regarding the injury prevention aspect of the program. Participants were simply asked to choose one of following: both stretching exercise and strength training (SM-group), only stretching exercise (S-group), only strength training (M-group), and neither stretching exercise nor strength training (N-group). The participants were asked to perform their selected exercise program once daily after baseball practice.

#### Self-stretching exercises for shoulder ROM deficits

To stretch the posterior inferior part of the dominant shoulder, we asked participants to perform a “sleeper stretch”^14^,^15^,^16^ with the dominant shoulder daily during baseball season (five repetitions of 60 seconds each). To perform this stretch, the participant lay on a firm surface with their scapula stabilized against the bed, their shoulder flexed 90°, their elbow flexed 90°, and their forearm in a neutral position. Then, they grasped their forearm proximal to the wrist with their opposite hand and passively internally rotated the dominant shoulder to its maximum ROM. This was held for one minute and repeated five times.

#### Shoulder external rotation strength training

To train external rotation strength, the participants were asked to lie prone on a bed with their dominant shoulder 90° abducted, dominant elbow 90° flexed, and dominant forearm in a neutral position (starting position) while holding a 500-ml plastic bottle filled with water (weight: approximately 500 g). They were instructed to rotate their dominant shoulder externally (concentric exercise) for 1 second and then return to the starting position (eccentric exercise) over 1 second. This movement was repeated 20 times (one set) followed by a short break; a total of three sets were performed.

### Data collection during baseball season

To ensure adherence to the prevention program and to detect when injuries occurred, the participants were asked to complete a daily self-recorded questionnaire regarding the presence of shoulder and/or elbow pain, performance of the self-stretching exercise and/or strength training, and limitations to pitching caused by shoulder and/or elbow pain. The questionnaires were filled out daily to avoid recall bias and were sent to us every month. In addition, we called the participants one to two times per month to encourage adherence to the prevention program, to confirm that they were completing the daily questionnaires, and to consult with them regarding their condition.

Based on their answers to the self-recorded questionnaire, a “shoulder or elbow injury” was defined as any condition resulting in the pitcher being considered disabled for ≥8 days^1^,^17^. Any injury that occurred via another mechanism, such as trauma from falls, collisions with other players, sprains while running, or being hit by a pitch, were excluded from the statistical analyses.

### Statistical Analysis

Statistical analyses were performed using SAS 9.4 (SAS Institute Inc., Cary, NC, USA). All tests were two-sided with a P = 0.05 significance level. Baseline characteristics are reported as means ± standard deviations. Group differences were evaluated using one-way ANOVA. Time-to-event curves were obtained using the Kaplan-Meier method and hazard ratios (HRs) for the occurrence of injury were calculated using multivariate Cox regression models. A log-rank test was used to compare the survival distributions among the groups.

*A priori* statistical power analysis indicated that 39 participants would provide a statistical power of 80% at an α level of 0.05 with a hazard ratio (HR) of 2.7^1^, an accrual interval of 150 days, a follow-up interval of 150 days, and a median time to failure in the group with the smallest time to failure of 50 days in the Kaplan-Meier analysis^18^. After the data were collected, *post hoc* power analysis was conducted to determine the statistical power of this study.

## Additional Information

**How to cite this article:** Shitara, H. *et al*. Shoulder Stretching Intervention Reduces the Incidence of Shoulder and Elbow Injuries in High School Baseball Players: a Time-to-Event Analysis. *Sci. Rep.* **7**, 45304; doi: 10.1038/srep45304 (2017).

**Publisher's note:** Springer Nature remains neutral with regard to jurisdictional claims in published maps and institutional affiliations.

## Figures and Tables

**Table 1:** Baseline characteristics of the study participants.

Baseline characteristics	N group (N = 14)		S group (N = 32)		SM group (N = 46)		*P* value
	Mean	SD	Mean	SD	Mean	SD	
Baseball experience (year)	7.9	2.3	8.1	2.0	8.3	1.8	0.76
Body height (cm)	174.8	4.1	172.4	6.0	172.7	4.8	0.34
Body weight (kg)	68.0	6.3	69.7	7.5	66.8	5.6	0.15
ABIR in dominant side (deg)	36.1	18.3	37.8	13.1	36.8	12.5	0.92
HA in dominant side (deg)	26.5	14.9	29.2	11.5	28.8	12.4	0.78
Elbow flexion in dominant side (deg)	144.1	6.8	144.1	4.5	143.6	4.6	0.88
Elbow extension in dominant side (deg)	2.9	6.5	2.1	6.2	2.8	7.2	0.90
PER in dominant side (lb)	24.2	5.3	24.6	6.7	23.7	5.1	0.77
PER ratio	1.0	0.2	1.0	0.2	1.0	0.2	0.98
PIR in dominant side (lb)	25.5	8.4	26.2	9.3	26.0	7.0	0.96
PIR ratio	0.9	0.2	1.0	0.2	1.0	0.2	0.22

ABIR: ROM of 90° abducted shoulder internal rotation, HA: horizontal adduction.

PER: prone external rotation strength, PIR: prone internal rotation strength.

Strength ratio = strength of the dominant side/strength of the nondominant side.

**Table 2:** Results of multivariate Cox regression models for hazard ratios.

Group	Total	Incidence of Injures	
	N	N (%)	HR (95% CI)
Non exercise	14	8 (57.1)	1.000
Stretching	32	8 (25.0)	0.355 (0.133–0.947)
Stretching + Muscle training	46	16 (34.8)	0.472 (0.201–1.106)

HR: Hazard Ratio, CI: Confidence Interval.
